# The Relation between the Rheological Properties of Gels and the Mechanical Properties of Their Corresponding Aerogels

**DOI:** 10.3390/gels4020033

**Published:** 2018-04-09

**Authors:** Mingze Sun, Hua Sun, Yutao Wang, Miguel Sánchez-Soto, David A. Schiraldi

**Affiliations:** 1 Department of Macromolecular Science and Engineering, Case Western Reserve University, Cleveland, OH 44106-7202, USA; mxs1031@case.edu (M.S.); hxs344@case.edu (H.S.); yutaowang528@hotmail.com (Y.W.); 2 Centre Catalá del Plástic, Universitat Politécnica de Catalunya, Barcelona Tech, C/Colom 114, 08222 Terrassa, Spain; m.sanchez-soto@upc.edu

**Keywords:** aerogel, rheology, mechanical properties

## Abstract

A series of low density, highly porous clay/poly(vinyl alcohol) composite aerogels, incorporating ammonium alginate, were fabricated via a convenient and eco-friendly freeze drying method. It is significant to understand rheological properties of precursor gels because they directly affect the form of aerogels and their processing behaviors. The introduction of ammonium alginate impacted the rheological properties of colloidal gels and improved the mechanical performance of the subject aerogels. The specific compositions and processing conditions applied to those colloidal gel systems brought about different aerogel morphologies, which in turn translated into the observed mechanical properties. The bridge between gel rheologies and aerogel structures are established in the present work.

## 1. Introduction

**Figure 1:**
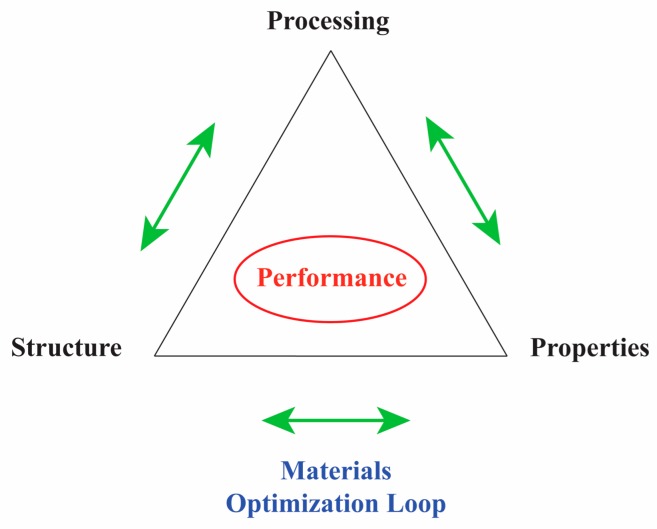
The strategy of studying materials science: the materials optimization loop.

The interdependence of processing, structure, properties, and performance of materials can be described using an optimization loop, as shown in Figure 1 [1,2]. The strategy in developing new materials, then, requires consideration of a multilevel structure, which leads a series of properties, characteristics, or behaviors in a certain environment, necessary to meet desire product performance [3].

Aerogels, low-density (typically 0.1 g/cm^3^ or less), porous materials that can be used in packaging, absorption, and insulation fields, have been intensely studied over the past twenty years and are typically produced using one of two processes, supercritical drying or freeze drying [4,5,6,7,8,9]. In the freeze drying process, the subject of this study, a hydrogel is frozen and ice is then removed via a sublimation process; the freezing of water largely dictates the morphology of the final aerogel, whose solid matter are concentrated within the grain boundaries of the ice [10,11]. By avoiding the melting of the ice, the inner structure collapse, due to capillary forces, is avoided [12]. It is not well understood how the rheological properties of the hydrogels, which clearly impact their viscoelastic properties, impact the final aerogel structure and properties. Understanding this relationship between rheology and product, for similar compositions, and its impact upon the product optimization loop, are the subjects of the present work [13,14,15]. Because of the polar nature of water and of smectite clays, freeze-dried aerogels are typically produced from polar polymers such as poly(vinyl alcohol) (PVOH), and ammonium alginate. Water allows for clay exfoliation, is environmentally desirable, and can be readily sublimed. Polymers of molecular weights that are readily soluble in a 2–10 wt % range are ideal for aerogel preparations. 

## 2. Results and Discussion

To assess hydrogel viscoelastic properties, and ultimately to bridge the gap between molecular structure and product performance, small deformation rheology experiments were conducted [16]. Measurements need to be carried out in the linear viscoelastic region to ensure that the sample properties are independent of imposed strain or stress. Oscillatory frequency sweep measurements, running under a constant oscillation amplitude (constant strain or stress) and constant temperature were carried out to determine the gels’ viscoelastic properties [15,17]. The samples’ viscoelastic parameters, such as the storage modulus (G’), the loss modulus (G”), the tangent of the phase angle (tanδ) and the modulus of complex viscosity (|η*|) (referred to as complex viscosity as a shorthand in this paper) were monitored and recorded against frequency. η* is a parameter based on the complex modulus (G*) (see Equation (1)), an overall resistance to deformation, and the integration of G’ and G” is written as Equation (2).

(1) $$\eta *=\frac{G*}{\omega }$$

(2) $$G*=G^{\prime }+{\mathrm{iG}}^{\prime \prime }$$

**Figure 2:**
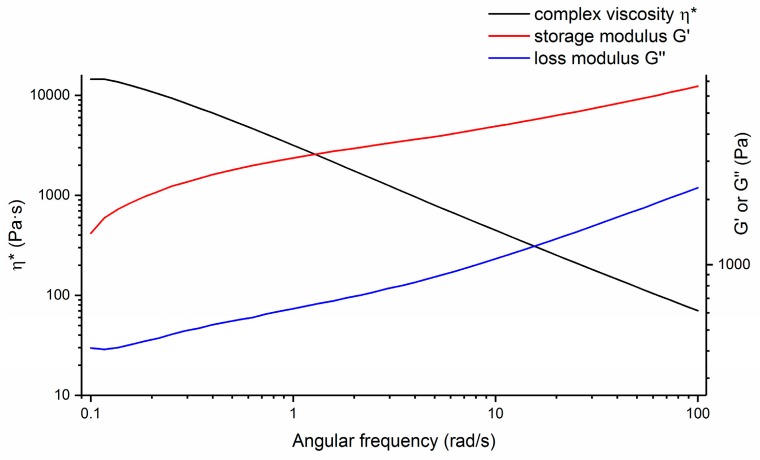
Oscillatory frequency sweep measurement on a representative hydrogel system: complex viscosity, elastic modulus, and loss modulus vs. frequency.

η*, because of this integration, is a good visible indicator of attributes such as the flexibility or stiffness of a material during formation. One more advantage of complex viscosity is that, when it is plotted as a function of angular frequency, it can be equated to a shear viscosity vs. shear rate profile for certain materials utilizing the Cox–Merz rule [18,19]. This process also provides a way of calculating the effective viscosity/shear rate profiles when it is hard to test effective viscosity from traditional methods. For the current system, hydrogel matrixes were tested, yielding the typical profiles as shown in Figure 2, which is a representative profile of a hydrogel matrix and G’ > G”, consistent with a matrix which behaves like an elastic solid [20]. No cross-over point between G’ and G” was observed, nor was there any region shift. The matrix behaved like a stable visco-elastic solid in the frequency range and exhibited behavior typical of soft gel or showed dispersion with a weak structure. Complex viscosity (η*) performance against frequency, therefore, was deemed important for studying the hydrogel’s rheological properties. Different hydrogel systems and process/composition variables were examined to bring about the complex viscosity changes; by monitoring η* performance, it is expected that the final product’s performance can be predicted without further fabrication.

### 2.1. Processing Rate

**Figure 3:**
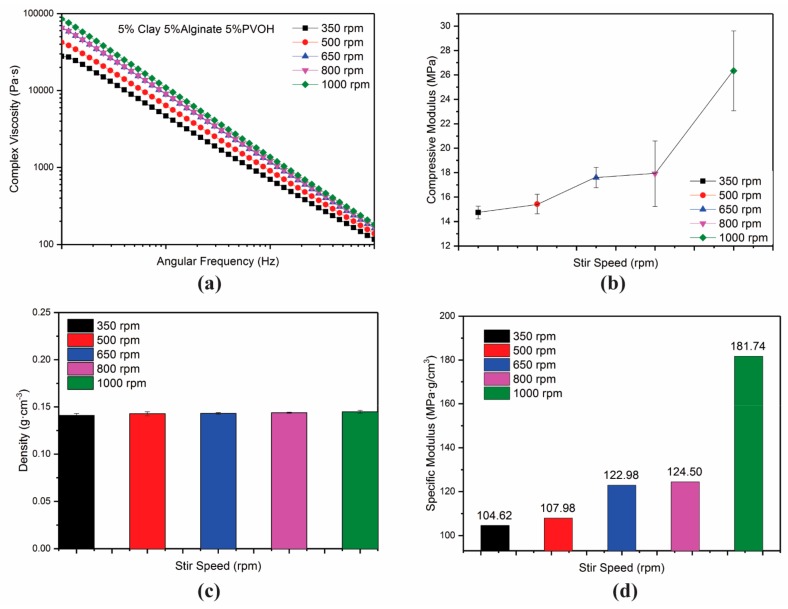
Viscosity of hydrogels (**a**); compressive moduli of respective aerogels (**b**); density vs. stirring speeds (**c**); specific modulus vs. stirring speeds (**d**).

Processing rate (in this case stirring speed), which controls dispersion of the hydrogel components, was the first factor examined, as it might impact rheological properties. The hydrogel system used here was 5% ammonium alginate/5% clay/5% PVOH (*M*w = 31 k). Ammonium alginate was selected to amplify the effect of viscosity due to the relative insensitivity of viscosity observed in the original clay–PVOH matrix system. Figure 3a shows that the hydrogel exhibited a general trend of increasing viscosity with stirring speed increase. Figure 3b shows that the modulus of aerogels behaves similarly to the stirring rate of hydrogel, and similarly increased as stirring speed increased. The viscosity of hydrogel stirred at 650 and 800 rpm performed similarly, which was also reflected simultaneously from a similar mechanical strength of the respective aerogel samples. All aerogels were produced from the same concentration and composition of the “alginate–clay–PVOH” matrix, which resulted in similar aerogel densities (see Figure 3c), and the specific modulus had the same performance as the overall modulus (see Figure 3d).

**Figure 4:**
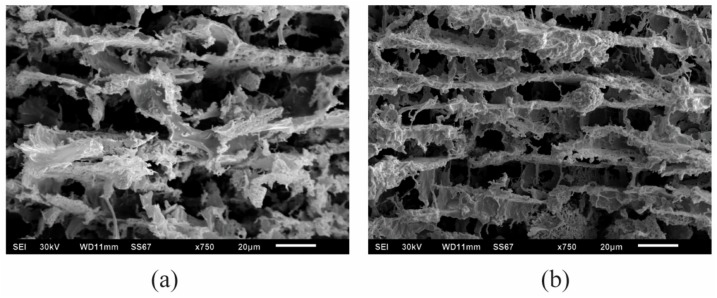
SEM images to show the difference inner structure morphology of aerogels made from same composition: 5% alginate/5% clay/5% PVOH with different stirring rates: 350 rpm (**a**); 1000 rpm (**b**).

The processing conditions that were examined above brought about a change in the viscosity and thus a change in the aerogel’s mechanical properties; the morphology of the aerogel’s inner structure undoubtedly become the bridge to link the former to the latter. Figure 4 presents SEM images of an aerogel sample produced from hydrogels stirred at 350 and 1000 rpm. From the inner structures, it is evident that the higher stirring speed mixed the matrix more thoroughly and coated the matrix more uniformly. As stirring speeds increase, molecular chains have a higher chance of entanglement, thus increasing viscosity. A greater number of layered structures were linked during mixing, bringing about to a more organized structure formation, with the associated increase in modulus.

### 2.2. Standing Times

The second factor evaluated was the standing time between the mixing and freezing of the hydrogels. The hydrogel samples were generally frozen immediately or shortly after stirring. The hydrogel system used in this part of work was 5% clay/2.5% PVOH (*M*w = 31 k). Fresh hydrogel was immediately frozen, or allowed to stand for 1 or 3 days before being frozen. The parts used for testing aerogel modulus were from the lower halves of the monoliths, and correspondingly a sample from the bottom of the hydrogel was retrieved for rheology testing.

**Figure 5:**
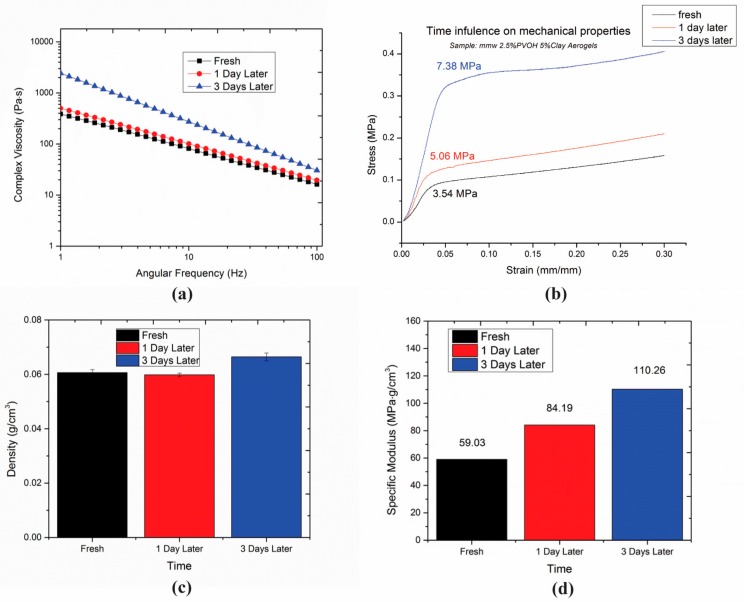
Related properties of hydrogels at various standing times. Viscosity (**a**), mechanical performance (**b**), different density (**c**), increasing trend of specific modulus (**d**).

**Figure 6:**
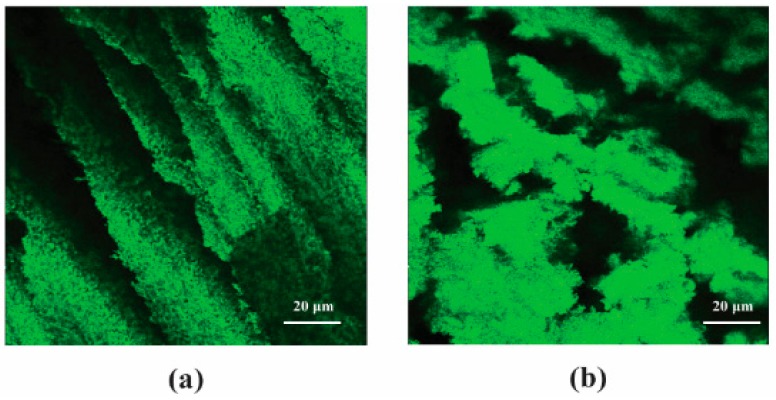
Confocal microscopy images showing different morphologies of aerogels made from hydrogel with various standing times: 0 days, frozen immediately (**a**); 3 days, frozen 3 days later (**b**).

Figure 5a,b show that the viscosities of the hydrogels and the moduli of the respective aerogels both increased as standing time increased. Different from the stirring speed effect, the density of the hydrogels changed as standing time increased (see Figure 6c), which implies that the composition changed at the bottom of the hydrogel. The specific modulus also increased, demonstrating that the modulus increase was not solely due to changes in density, Figure 5d.

Densities and compositions within an aerogel monolith can vary from top to bottom, as a result of clay sedimentation. Since the hydrogels are highly viscous suspensions, after standing for a sufficient period of time, clay sediments can form a gradient due to gravity. The bottom portion of an aerogel from a hydrogel allowed to stand for 3 days therefore exhibited a higher density. The structure of the lower part enhanced due to additional clay, causing a higher modulus and specific modulus. Based on confocal images (Figure 6), the aerogels from the immediately frozen samples kept the classic lamellar structure, while samples fabricated after 3 days exhibited a stack of clay deposition [21,22,23,24]. The aerogel modulus increased as a result of the matrix stack effect. After a longer time, such as a week, structures were no longer organized and collapsed inside, which resulted in poor mechanical performance.

### 2.3. Polymer Molecular Weights

**Figure 7:**
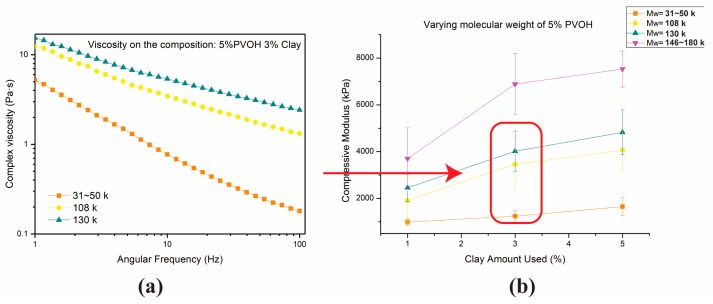
The viscosity properties of hydrogels of the same composition prepared with various Mw PVOH solutions(**a**) as well as the mechanical performance of the respective aerogel products (**b**).

The molecular weight of PVOH used in this study is discussed in this section. The hydrogel system used for this comparison was 3% clay/5% PVOH (varied *M*w). The viscosity of the hydrogel system can be tuned by changing the molecular weight of PVOH. Figure 7 shows that the viscosity increased as PVOH molecular weight increased, resulting in an aerogel modulus increase, similar to the conclusions of Lamison et al. [25]. From the view of viscosity, a higher Mw of PVOH means greater chances for local polymer chain entanglement. Even without the cooperation of clay, the preparation of PVOH solutions with different Mws can still bring about this phenomenon: as the molecular weight of PVOH increases, the respective PVOH solutions become more difficult to prepare.

**Figure 8:**
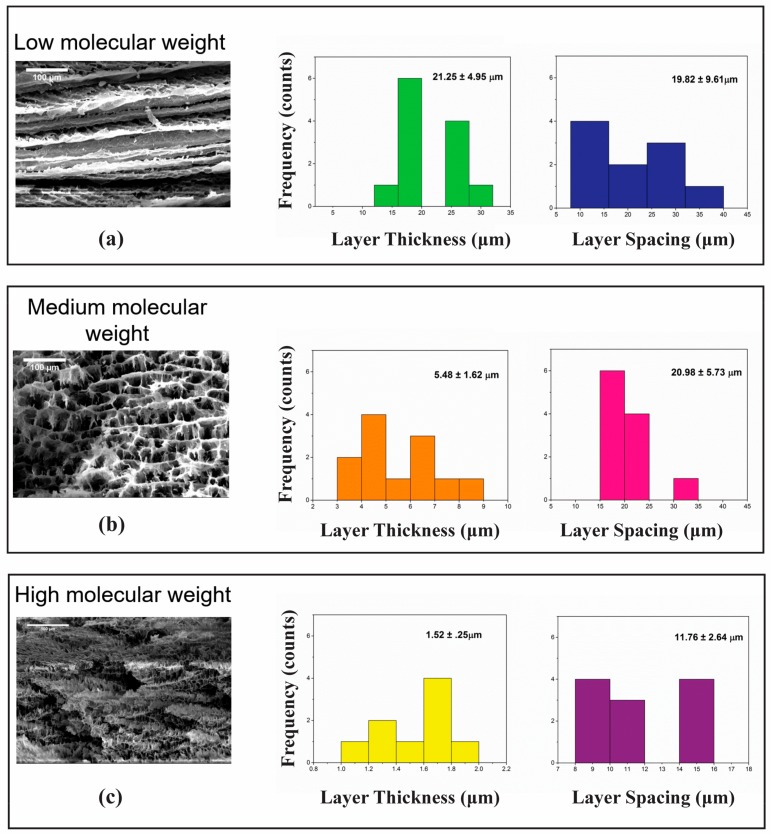
A series of SEM images as well as the layer thickness and spacing, exhibiting the influence of hydrogel viscosity based on different Mw values of PVOH. (**a**) Incorporated with low molecular weight (31–50 k) PVOH; (**b**) Incorporated with medium molecular weight (108 k) PVOH; and (**c**) Incorporated with high molecular weight (146–180 k) PVOH.

When mixing with PVOH solutions with higher *M*ws, the system viscosities inevitably increased, which led to a granular layer structure formation when the hydrogels were frozen. Figure 8 displays a series of aerogel internal morphologies and related layer thicknesses and layer spacings. All aerogels prepared in this comparison study were based on 5% clay/2.5% PVOH. The only difference in this study was the molecular weight of the PVOH: (a) 31–50 k, low *M*w; (b) 108 k, medium *M*w; (c) 146–180 k, high *M*w. All aerogels in this comparison exhibited a layered structure and small pores in each layer (Figure 8a–c SEM images). These pores are attributed to the polymer–clay–water interaction during ice crystallization, which hinders ice growth when it is frozen into large platelets (a lamellar structure); due to the difficulty of pushing the matrix aside during ice platelets formation, as viscosity in the matrix increases, ice crystallization increases. SEM images show increasing pores generated with increased viscosity. The layer thicknesses and layer spacing counts measured via SEM images also demonstrate that thickness and spacing decreased due to a greater number of pores, which was finally attributed to the high-viscosity system. The greater number of pores and granular structure formation from high-viscosity matrixes can also provide insight on the fabrication of samples used for thermal insulation, using the special granular structures to capture more air and hinder the heat flow transfer.

## 3. Conclusions

This work has demonstrated that the viscosity of hydrogels of similar compositions, which were later freeze-dried to form aerogels, impacts the aerogel structure and mechanical properties. Through tuning different factors such as processing rate, standing time, and the Mw of the polymers used, similar performances were observed, and a qualitative relationship between the viscosity of hydrogels and the modulus of aerogels was successfully established; thus, one can predict the properties of an aerogel based on its hydrogel viscosity alone, thereby saving time and energy.

## 4. Experimental

### 4.1. Materials

Sodium montmorillonite (PGW grade; Nanocor Inc., Arlington Heights, IL, USA), poly(vinyl alcohol) (PVOH, *M*w ≈ 31,000~50,000 “low *M*w”; 130,000 “high *M*w”; 146,000~180,000 “ultra high *M*w”; 99+% hydrolyzed, Sigma Aldrich (St. Louis, MO, USA), as well as *M*w ≈ 108,000 “medium *M*w”; 99.7% hydrolyzed and a polydispersity index of PDI ≈ 1.7, Polysciences, Inc., Warrington, PA, USA), and ammonium alginate (Pfaltz & Bauer, Waterbury, CT, USA) were all used as received. Deionized water was prepared using a Barnstead RO pure low-pressure, reverse osmosis system (Thermo Fisher Scientific, Marietta, OH, USA). 

### 4.2. Hydrogel Preparation

**Figure 9:**
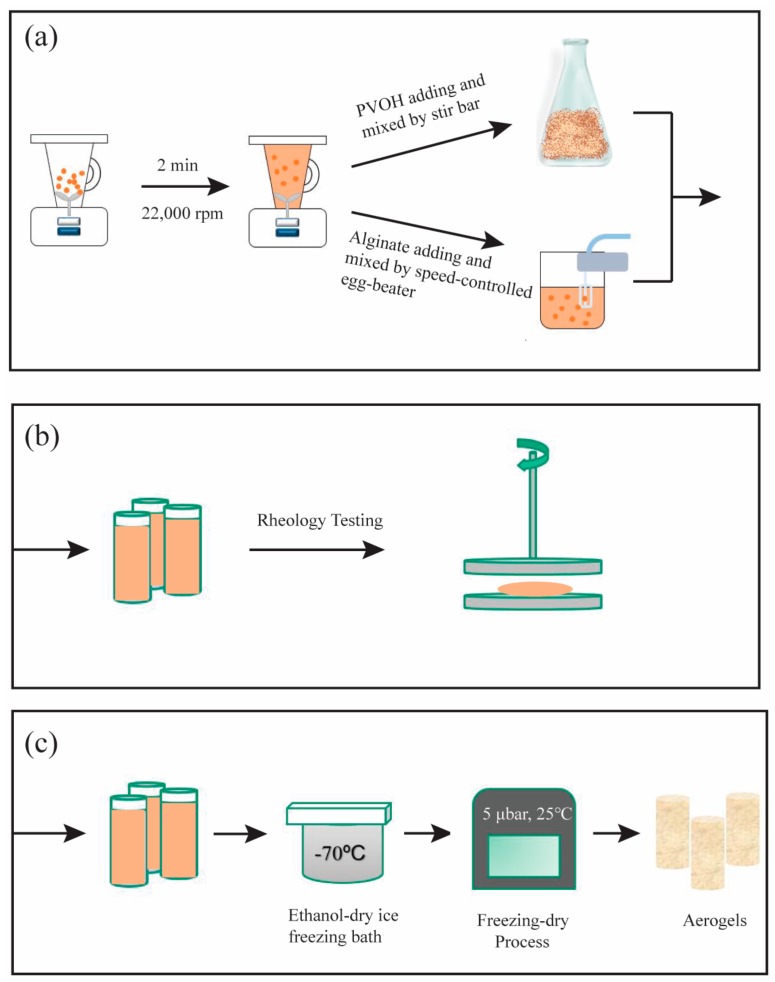
Illustration of aerogel fabrication and rheology test. Hydrogel preparation through high speed blender in order to ensure a good dispersion of clay suspension. And a gentle mixing with PVOH in order to avoid bubbles generated. Egg beater which can be controlled mixing rate was used to mix alginate(**a**); Once completed hydrogel preparation, specimen could be sent for a rheology test (**b**) or went through freeze-drying process to fabricated aerogels (**c**).

Loadings of clay, PVOH, and alginate were given as percentages, based on mass of DI water employed. For example, in order to formulate a hydrogel containing 5 wt % clay and 5 wt % PVOH, 200.0 mL of deionized water and 20.0 g sodium montmorillonite were placed into a 1.0 L blender and then mixed at a high speed (22,000 RPM) for approximately 2 min to wet the clay completely and create a 10 wt % clay hydrogel. Two hundred milliliters of a 10 wt % PVOH solution was then added slowly at a low speed mixing to create the clay/polymer hydrogel with 5 wt % clay and 2.5 wt % PVOH. For aerogels that contained 5% ammonium alginate, appropriate amounts of raw materials, for example, 20 g, were added to the 400 mL hydrogel made above, which was then mixed with an egg-beater with various speed tuning through voltage transformer, to obtain alginate/clay/polymer gels comprising 5 wt % alginate, 5 wt % clay, and 5 wt % PVOH. This procedure is illustrated in Figure 9a.

### 4.3. Viscosity Testing

The complex viscosities of hydrogel specimens were measured using an advanced rheometric expansion system (ARES) strain controlled rheometer (TA instruments, New Castle, DE, USA. Hydrogel specimens of 1–2 mL were carefully loaded between two parallel plate fixtures, in which the bottom plate is fixed and the top one rotates with an imposed torque. The test was set to run an oscillation frequency sweep from (0.1 to 100 rad/s), with a preset constant amplitude. Before starting a test, the excess gel was removed to ensure a clear and smooth interface; results are plotted as G’, G’’, or complex viscosity vs. frequency, as illustrated in Figure 3b.

### 4.4. Aerogel Formation

Approximately 12 mL samples of the mixtures were transferred into 5 dram (18.5 mL) polystyrene vials and subsequently frozen by immersing the vials into a solid carbon dioxide/ethanol cooling bath (−80 °C). The frozen samples were then dried in the VirTis Advantage EL-85 freeze dryer (SP Scientific, Warminster, PA, USA, which has a shelf temperature of 25 °C and an ultimate chamber pressure of 5 μbar to sublime the ice. The entire freeze drying process required 72–96 h to insure completion of the process, as shown in Figure 9c.

### 4.5. Mechanical Testing

Compression testing was conducted on cylindrical aerogel samples (measuring about 20 mm in diameter and 20 mm in height), using an Instron model 5565 universal testing machine (Instron, Norwood, MA, United States, fitted with a 1 kN load cell. These tests were conducted at a constant strain rate of 10 mm/min. Five to eight samples for each batch with various compositions were tested for reproducibility. The initial compressive modulus was calculated from the slope of the linear area, obtained from the initial part of the stress–strain curve.

### 4.6. SEM and Confocal Microscopy

Morphological microstructures of the aerogels were observed using a JEOL scanning electron microscope (JSM-IT100, JEOL, Peabody, MA, USA). The samples were pre-treated by fracturing in liquid nitrogen then sputter coated with 5 nm gold before testing. In order to observe the inner structure as a 3D view, a Leica confocal laser microscope was applied to display the inner morphology without breaking samples inside. The specimens were pre-cut by razor and double-taped on the glass slide in order to load for scanning.

## Abbreviations

G’	storage modulus
G’’	loss modulus
G*	complex modulus
η*	complex viscosity
PVOH	poly(vinyl alcohol)
*M*w	molecular weight
